# Borderline Personality Disorder: An Evidence-Based Guide for Generalist Mental Health Professionals

**DOI:** 10.1192/pb.bp.113.044677

**Published:** 2014-10

**Authors:** Rhiannon Pugh

**Figure:**
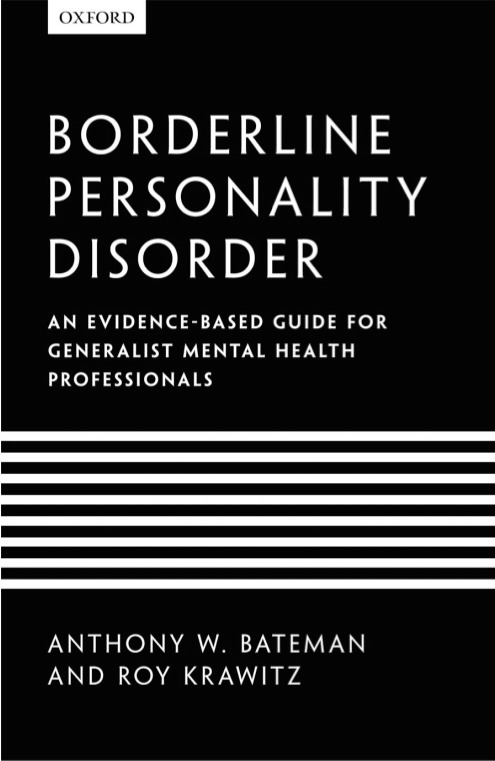


A 350-word review is not enough to do this book justice. Written by two psychiatrists, one with a psychodynamic and the other a behavioural orientation, the book succeeds in outlining different psychological and pharmacological approaches to the treatment of borderline personality disorder in a harmonious and enlightened way.

This book is in part a spin-off from a research project comparing mentalisation-based therapy with structured clinical management. The latter proved strikingly effective as a comparator treatment for borderline personality disorder and much of this book describes the components of this approach. It contains, however, far more than this.

The book is written with compassion and a clear commitment to working with this patient group, and as well as being comprehensively researched it is accessible and easy to read. There are eight chapters, each with a helpful summary.

For the busy clinician, I would recommend the sections on crisis management and planning, self-harm and chain analysis. For those with a bit more time, many chapters stand alone as a good read - I found chapter one, a comprehensive overview of borderline personality disorder, and chapter six, on prescribing and in-patient management, particularly helpful. Throughout there are plenty of practical tips, ‘consumer comments’ that bring the clinical picture of borderline personality disorder to life, and an excellent finale: ‘top 10 additional resource-efficient treatment strategies’, which is a great addition to psychological strategies that are discussed in more depth in the rest of the book.

The book is generous in its scope and is clearly written to be shared with patients, their relatives and their friends. The chapter on their involvement is packed full of useful information and I was impressed by the clever use of editing, whereby written materials suitable for copying and distributing are printed on separate pages for ease of use.

This book is highly recommended and I consider the authors to have achieved what they set out to do: instil hope in all of us working with patients with borderline personality disorder and, in so doing, instil hope in patients themselves.

